# Comparative transcriptomes reveal geographic differences in the ability of the liver of plateau zokors (*Eospalax baileyi*) to respond and adapt to toxic plants

**DOI:** 10.1186/s12864-023-09642-5

**Published:** 2023-09-06

**Authors:** Yuchen Tan, Yanli Wang, Qianqian Liu, Zhicheng Wang, Shangli Shi, Junhu Su

**Affiliations:** 1 College of Grassland Science, Key Laboratory of Grassland Ecosystem (Ministry of Education), Gansu Agricultural University, Lanzhou, 730070 China; 2 Gansu Agricultural University-Massey University Research Centre for Grassland Biodiversity, Gansu Agricultural University, Lanzhou, 730070 China

**Keywords:** Comparative transcriptome, Plateau zokor, *Stellera chamaejasme*, Detoxify, Adaptive capacity

## Abstract

**Background:**

Environmental changes are expected to intensify in the future. The invasion of toxic plants under environmental changes may change herbivore feeding environments. Herbivores living long-term in toxic plant-feeding environments will inevitably ingest plant secondary metabolites (PSMs), and under different feeding environments are likely to have unique protection mechanisms that support improved adaptation to PSMs in their habitat. We aimed to compare different subterranean herbivore population responses and adaptations to toxic plants to unveil their feeding challenges.

**Results:**

Here, we investigated the adaptive capacity of the liver in two geographically separated populations of plateau zokors (*Eospalax baileyi*) before and after exposure to the toxic plant *Stellera chamaejasme* (SC), at the organ, biochemical, and transcriptomic levels. The results showed no significant liver granules or inflammatory reactions in the Tianzhu (TZ) population after the SC treatment. The transaminase level in the TZ population was significantly lower than that in the Luqu population. Transcriptome analysis revealed that the TZ population exhibited interactions with other detoxification metabolic pathways by oxytocin pathway-associated genes, including diacylglycerol lipase alpha (*Dagla*), calcium/calmodulin dependent protein kinase II Alpha (*Camk2a*), and CD38 molecule (*Cd38*). The phase II process of liver drug metabolism increased to promote the rate of metabolism. We found that alternative splicing (AS) and the expression of the cyclin D (*Ccnd1*) gene interact—a TZ population hallmark—reduced liver inflammatory responses.

**Conclusion:**

Our study supports the detoxification limitation hypothesis that differences in liver detoxification metabolism gene expression and AS are potential factors in herbivore adaptation to PSMs and may be a strategy of different herbivore populations to improve toxic plant adaptability.

**Supplementary Information:**

The online version contains supplementary material available at 10.1186/s12864-023-09642-5.

## Introduction

The severity of environmental change caused by human activities is increasing (IPCC, 2022) [1]. For example, droughts resulting from climate change can precipitate the degradation of grasslands, the reduction in vegetation diversity, and the decline in food selectivity and availability among herbivores [2–4]. Additionally, toxic weeds quickly become the dominant species in the process of grassland degradation, increasing the difficulty for herbivores to forage. This, coupled with food intake simplification, can lead to an increase in toxins from plant secondary metabolites (PSMs) in herbivore diets [5, 6]. Therefore, changes in the quantity and quality of vegetation, as well as the emergence of PSMs, present considerable obstacles to the health and survival of local herbivores [1, 7]. The unique metabolic mechanisms of herbivores that have fed on poisonous plants may provide more survival opportunities and lead to the development of unique protection mechanisms [8]. Understanding how herbivores adapt to PSMs toxins in their habitats under different environmental conditions can improve our understanding of their responses to environmental changes.

The detoxification–limitation hypothesis states that herbivores understand the negative effects of toxic plants, decrease these effects after weighing the risks, and maximize their nutritional benefits [9]. Herbivores reduce their intake of PSMs through migration, antifeedant behavior, or broadening their diet [10]. However, herbivores with limited migratory abilities cannot rely on antifeeding behaviors to avoid PSMs. Therefore, physiological and biochemical metabolic adaptability becomes a more effective option to compensate for food shortage challenges [11]. When herbivores ingest PSMs, pressure is exerted on the liver for detoxification [12, 13]. In the species *Neotoma lepida* and *N. bryanti*, a notable increase in the expression of the Cytochrome P450 Monooxygenase (P450) gene was observed subsequent to the consumption of PSMs, which is closely associated with liver detoxification [14, 15]. Monarch butterflies (*Danaus plexippus*) inhabiting the southwestern United States display gene expression alterations in response to varying concentrations of milkweed toxins (cardenolides). In addition to an up-regulation of conventional liver detoxification genes, these butterflies exhibited a down-regulation of genes associated with immune function, potentially alleviating the oxidative stress caused by PSMs [16, 17]. This adaptation to toxic plants allows herbivores to adapt to environmental changes and new ecological niches.

The toxic plant feeding experiences for herbivores may result in different metabolic patterns for ingested PSMs [12, 18]. Specialist herbivores are often more efficient than generalists at processing PSMs [19, 20]. Stephen’s woodrats (*N. stephensi*) showed increased detoxification of the liver (*Cyp2b*) gene after consuming juniper (*Juniperus monosperma*) compared to the reaction of white-throated woodrats (*N. albigula*) [21]. This suggests that herbivores can adjust their metabolic adaptive capacity for detoxification in response to PSMs under varying environmental conditions. However, not all herbivores have adapted to PSMs by altering gene expression. Stephen’s woodrats can induce the production of one or multiple CYP2B variants, thereby augmenting the hepatic reaction to the α-pinene metabolic rate and enhancing the woodrats’ capability to detoxify and metabolize PSMs [22]. Jacobs et al. (2021) suggested that alternative splicing (AS) events in different populations may mediate adaptation differences, which deserves more attention as a potential mechanism for adapting to environmental changes [23, 24]. These studies further emphasize the diverse response strategies of herbivores to PSMs under different environmental conditions. Many previous studies in this field have focused on individual species within a single biological hierarchy. Therefore, further research at the broader scale on how herbivores adapt to PSM toxins under different environmental conditions can deepen our knowledge in this context.

The plateau zokor (*Eospalax baileyi*) is a typical subterranean herbivore that survives in burrows most of the year under harsh environmental conditions, obtains food through digging, and has a weak migratory ability [see Additional file 1] [25]. Owing to disturbances from human activities and environment changes, toxic weeds have become widespread with the toxic plant *Stellera chamaejasme* (SC) becoming a dominant grassland species [26]. SC has thick rhizomes with a high nutrient content [see Additional file 1]. According to optimal foraging theory, it has a high-cost performance and is consumed by the local plateau zokor, accounting for approximately 5% of their daily food intake [27]. Conversely, areas with less environmental change and grassland degradation have fewer of these plants. Plateau zokor populations in these areas have a wide variety of food but lack SC feeding experience, and under different geographical conditions undergo genetic differentiation due to spatial isolation, with differences in feeding width [28]. It is likely that two plateau zokor populations in different geographic environments may have different metabolic capacities owing to their different SC feeding experiences. This renders them effective in models studying herbivore response strategies to PSMs under different degrees of environmental change. Therefore, to improve our knowledge in the field of herbivore differences in PSM adaptation, we investigated differences in liver responses to PSM toxins in plateau zokor from different geographic populations.

Here, we examined changes in the oxidative stress, transaminase indices, and liver transcriptomes between different geographic populations under SC treatment in laboratory-controlled conditions. The focus was on the genes and pathways involved in liver detoxification and metabolism in different populations. We also examined the enrichment of other pathways involved in differentially expressed genes (DEGs) and determined whether plateau zokors have their own way of responding to the effects of SC. Transcriptome comparisons will help to deepen our understanding of the response strategies of different herbivore populations against toxic plants and to assess the differential responses of gene expression and adaptive capacity to PSMs.

## Results

### Effects of SC on histopathology

**Fig. 1:**
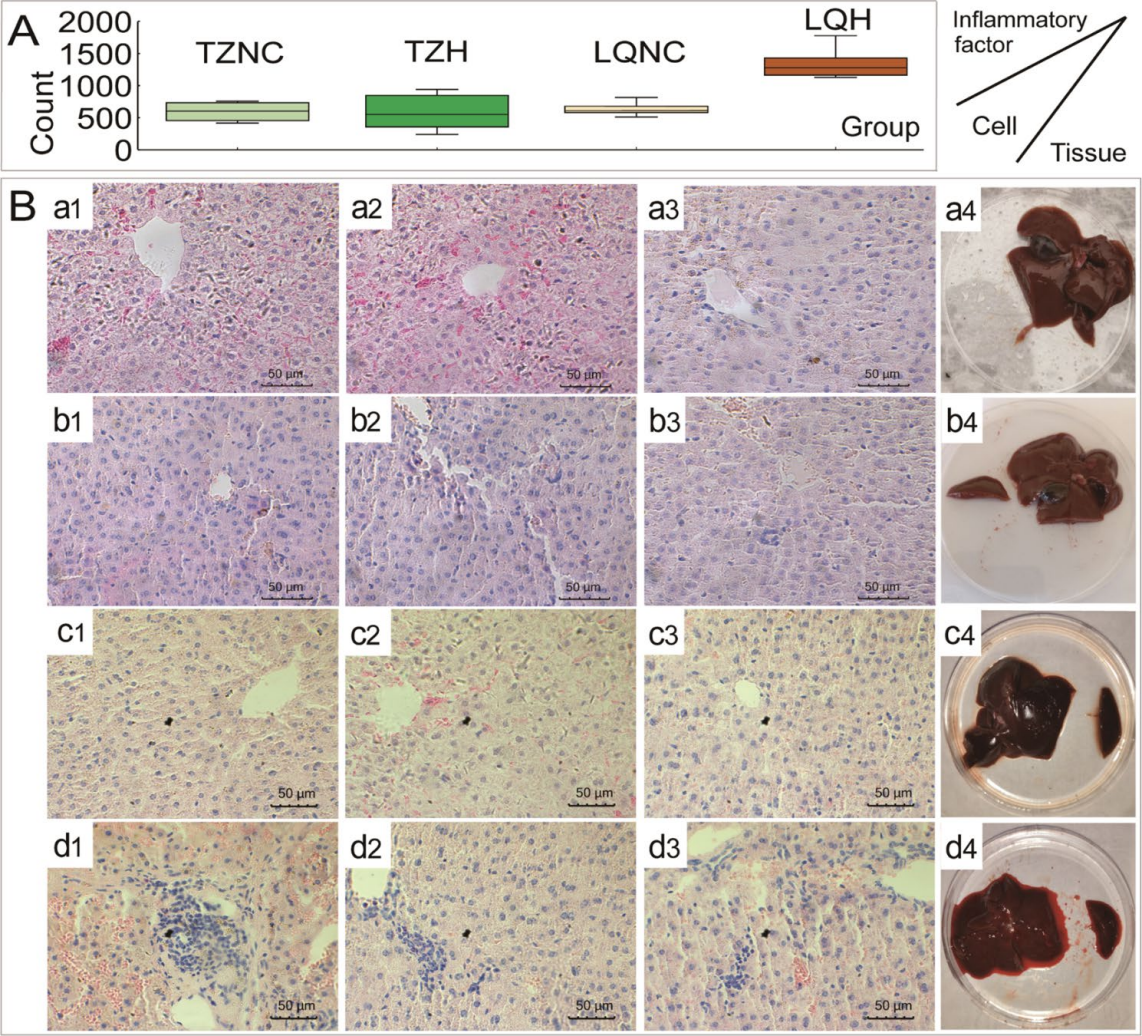
The effect of *Stellera chamaejasme* water extract treatment on the hepatic pathology of plateau zokor (*Eospalax baileyi*). Number of inflammatory cells in the hepatic tissue of plateau zokor (**A**). Plateau zokor hepatic tissue and microstructure (**B**). Tianzhu normal control group (TZNC) hepatic microstructure (a1, a2, and a3), liver organ (a4); Tianzhu high dose group (TZH) hepatic microstructure (b1, b2, and b3), liver organ (b4); Luqu normal control group (LQNC) hepatic microstructure (c1, c2, and c3), liver organ (c4); Luqu high dose group (LQH) hepatic microstructure (d1, d2, and d3); and liver organ (d4). Scale bar = 50 μm

There was no notable difference in liver histopathology between different populations of plateau zokor before the SC treatment (Fig. 1B, a1-a2, c1-c2). We found that different populations of plateau zokor exhibited different liver inflammation levels after the SC treatment. The number of inflammatory cells in the hepatic tissue of the LQ population after treatment was double that of the TZ population (Fig. 1A, *P* < 0.05). The amount of hemorrhage in the hepatic tissue of the LQ population was substantially higher than that in the TZ population (Fig. 1B, b2 and d2). The LQ population treated with SC showed hepatocyte necrosis, inflammatory cell aggregation, and scattered aggregates of lymphocytes in both the portal vein and hepatic lobules. The hemolysis of hepatic cells occurred, and intercellular erythrocyte lysis increased. Microscopic reddish granules appeared in the cytoplasm of cells in the hepatic lobules (Fig. 1B, d1–d2). However, no severe inflammatory responses were observed in the hepatic tissue of the TZ population (Fig. 1B, b1–b2).

**Fig. 2:**
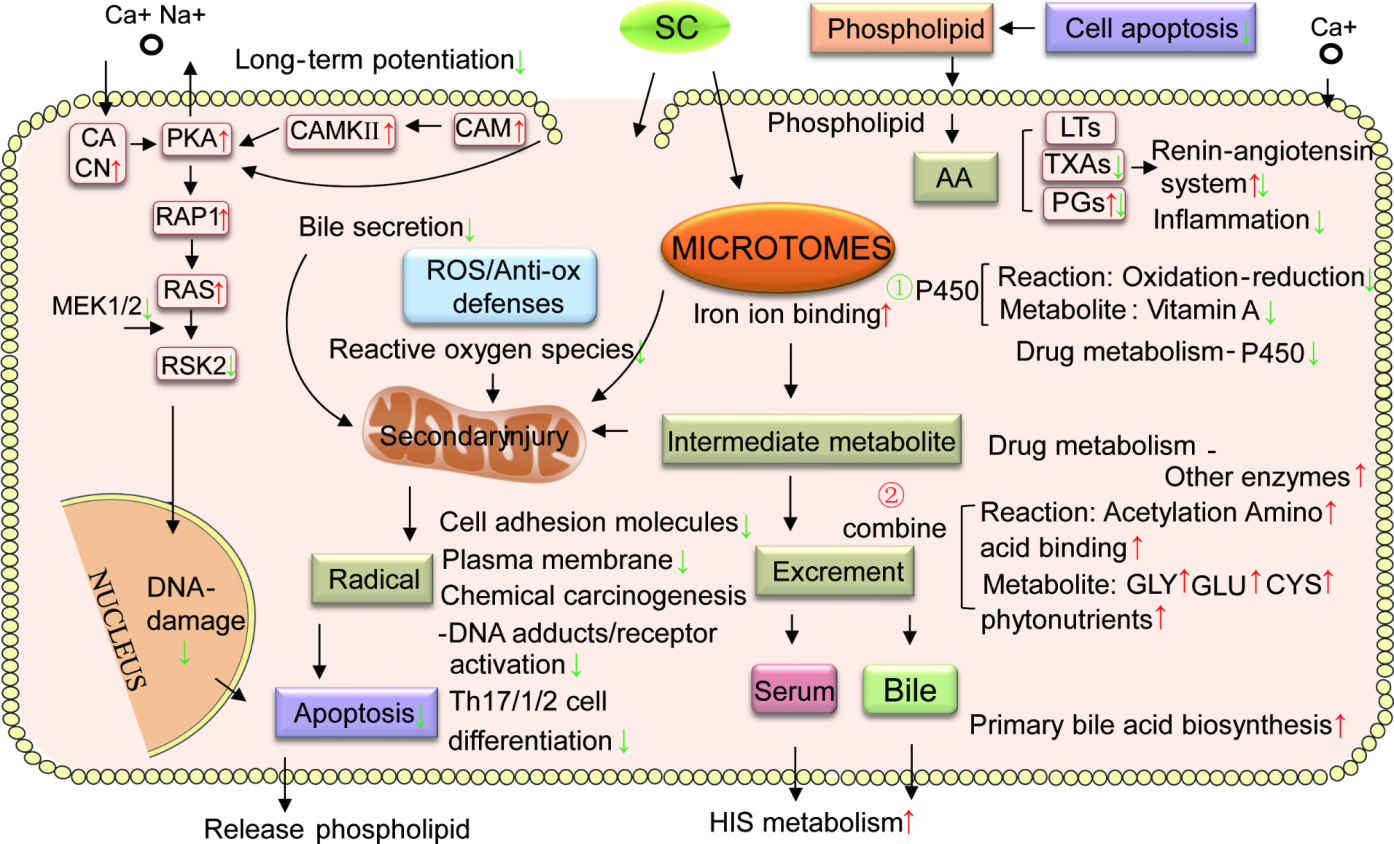
A conceptual flow diagram of SC metabolism in the hepatic cells of the TZ population. *Stellera chamaejasme* (SC) are endocytosed into the cell and vacuoles (green) in hepatic cells (pink). The key processes (dark pink) are involved in the response and metabolism of the liver to SC. The arrows represent upregulated genes or pathways in TZH relative to LQH, and the green arrows represent downregulated genes or pathways in TZH relative to LQH. CACN, calcium voltage-gated channel auxiliary subunit alpha 2 delta 2; PKA, protein kinase camp-activated catalytic subunit alpha; CAMKII, calcium/calmodulin-dependent protein kinase II alpha; CAM, calmodulin-like 4; RAP1, ras-related protein 1a; RAS, hras proto-oncogene, GTPase; RSK2, ribosomal protein S6 kinase A2; MEK1/2, Mitogen-activated protein kinase kinases 1 and 2; LTs, leukotrienes; TXAs, thromboxanes; PGs, prostaglandins; GLY, glycine; GLU, glutamic; CYS, cysteine

### SC treatment effects on blood biochemical indices

There was no notable difference in blood biochemical indices between different populations of plateau zokor before the SC treatment. We used ANOVA to compare the levels of AST (21.10 ± 4.95 U/L) and ALT (34.91 ± 1.36 U/L) observed in the TZ population following the treatment. The LQ population exhibited significantly higher levels of AST (43.81 ± 0.95 U/L) and ALT (57.07 ± 5.16 U/L) (*P* < 0.05). However, there was no statistically significant difference between the different populations regarding the content of MDA (158.20 ± 16.95 and 169.70 ± 6.42 nmol/ml) after the treatment (*P* > 0.05) (Table 1). This indicated that the oxidative stress function of the plateau zokor had an abnormal response to the SC treatment, and that there was a noticeable hepatic impairment response in the LQ population.

**Table 1:** Blood physiological and biochemical indices in plateau zokor treated with *Stellera chamaejasme*

	TZNC	LQNC	TZH	LQH
ALT (U/L)	34.95 ± 1.01^b^	34.58 ± 5.56^b^	34.91 ± 1.36^b^	57.07 ± 5.16^a^
AST (U/L)	13.07 ± 5.34^b^	10.94 ± 2.23^b^	21.10 ± 4.95^b^	43.81 ±. 95^a^
MDA (nmol/ml)	120.75 ± 25.73^b^	130.75 ± 5.11^ab^	158.20 ± 16.95^ab^	169.70 ± 6.42^a^

(A) alanine aminotransferase (ALT), (B) aspartate aminotransferase (AST), and (C) malondialdehyde (MDA) levels in plateau zokors treated with and without SC. Different letters between columns indicate significant differences between groups. Statistical analysis for outcomes with groups was conducted using one-way ANOVA followed by Duncan’s post-hoc test (*P* < 0.05). TZ normal control (TZNC), LQ normal control (LQNC), TZ high dose (TZH), and LQ high dose (LQH)

### Transcriptome responses of plateau zokor to the SC treatment

#### Responses of plateau zokor to SC hepatic differentially expressed genes

There were no notable differences in gene expression between TZ and LQ populations before SC treatment. However, after SC treatment, substantial differences in gene expression were observed between the two populations. Moreover, we observed certain genes that exhibited considerable differences before the SC treatment but no notable differences after the treatment. There were 3204 differential, 1399 upregulated, and 1795 downregulated genes identified between different populations. Interferon-induced transmembrane protein 2 (*Ifitm2*), transcription factor-like 5 (*Tcfl5*), and CKLF-like marvel transmembrane domain containing 7 (*Cmtm7*) genes were found and are involved in transmembrane transport, with increases in expression of 60-fold, 41-fold, and 13-fold, respectively. Transient receptor potential cation channel subfamily V member 4 (*Trpv4*) genes involved in ion transport showed a significant increase of 37-fold (*P* < 0.05) [see Additional file 2].

We performed a gene ontology (GO) function enrichment analysis of DEGs between different populations of plateau zokor after the SC treatment [see Additional file 1 and 3]. In addition to the enrichment of glutathione metabolism, proximal tubule bicarbonate recovery, catabolic gene molecular function before treatment [see Additional file 1], immune response and system process, ion binding, enzyme activity, and receptor binding molecular functions were significantly enriched (*P* < 0.05) [see Additional file 1 and 3].

We performed a KEGG enrichment analysis of DEGs between different populations of plateau zokor after the treatment [29–31]. In addition to glycine, we found serine and threonine metabolism and biosynthesis pathways in different populations of plateau zokor after treatment [see Additional file 1]. Retinol metabolism, chemical carcinogenesis-DNA adducts, and cytochrome P450 pathways—such as xenobiotic metabolism, steroid hormone biosynthesis, and drug metabolism-cytochrome—were significantly enriched after treatment (*P* < 0.05) [see Additional file 1 and 4].

### Plateau zokors respond to SC by regulating inflammatory pathways

**Fig. 3:**
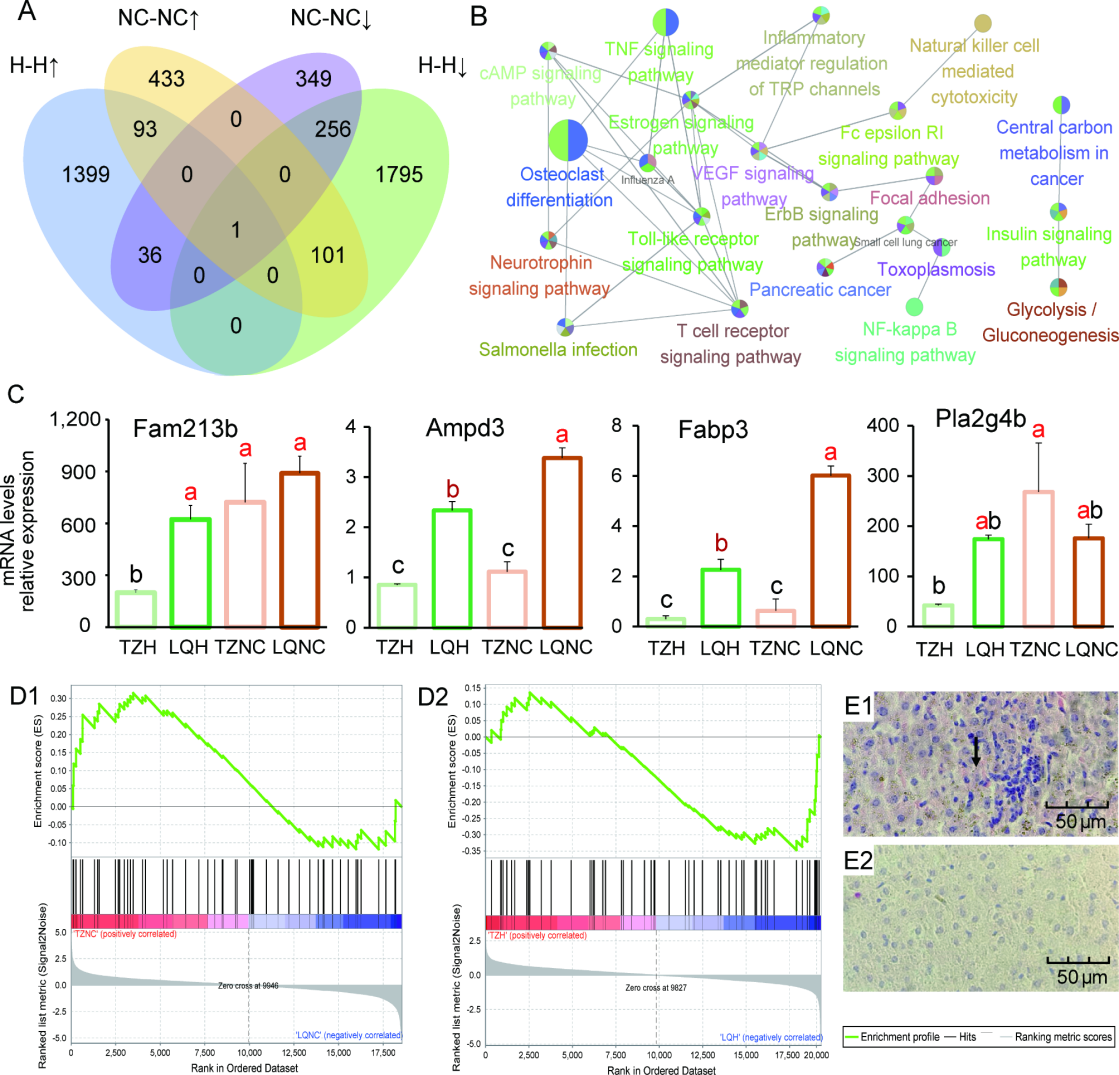
Different metabolic pathways in the liver of plateau zokor in different regions under SC treatment. (**A**) Identification of DEGs in the TZ and LQ populations before and after SC treatment. A Venn diagram showing an overlap in the numbers of DEGs between the four comparisons (TZNC vs. LQNC downregulated genes, TZNC vs. LQNC upregulated genes, TZH vs. LQH downregulated genes, and TZH vs. LQH upregulated genes). (**B**) KEGG pathway interaction network in the unique downregulated genes of the TZ population compared to the LQ population after SC treatment. (**C**) Expression profiles of genes involved in post-translational modifications and chaperones. Letters above the bars indicate significance (ANOVA, *P* < 0.05). TZH, LQH, TZNC, and LQNC are shown in light green, dark green, light yellow, and orange, respectively. (D1 and D2) GSEA analyses of gene sets for TZ (top) and LQ (bottom) populations after SC treatment for arachidonic acid (AA) pathway development. Positive and negative ES values indicate higher and lower expressions in the TZ population, respectively. Blue and red are low and high levels, respectively. (E1 and E2) The degree of liver inflammation in the LQ and TZ populations. Arrows indicate aggregation of inflammatory factors

We performed a cluster analysis of gene expression and found that there were only a few overlapping genes in different populations of plateau zokors after the SC treatment (Fig. 3A). We observed that unique downregulated genes were enriched into inflammatory and cancer-related pathways in the TZ population after treatment (Fig. 3B). This was a potential response factor for the smaller hepatic inflammatory response in this population. Among these responses, the expression of the nudix hydrolase 1 (*Nudt1*) gene—involved in purine metabolism and encoding a proteolytic enzyme that metabolizes phosphate caused by DNA damage—downregulated 48-fold. The expression of the adenosine monophosphate deaminase 3 (*Ampd3*) gene downregulated 2-fold, promoting cancer cell differentiation. The expression of the fatty acid binding protein 3 (*Fabp3*) gene downregulated 15-fold, reduce organ damage (Fig. 3C).

In gene set enrichment analysis (GSEA) of DEGs, the arachidonic acid (AA) pathway underwent a significant downregulation in the TZH population (*P* < 0.05) (Fig. 3, D1, and D2). The key gene responsible for this regulation—phospholipase A2 group IVB (*Pla2g4b*)—show was downregulated by 4-fold in expression, and the reduction of its encoded phospholipase A2 (cPLA2) blocked the formation of AA (*P* < 0.05) (Fig. 3C). We have previously demonstrated that the LQ population exhibited AA pathway upregulation associated with inflammation in response to the SC treatment [32]. In this result, the inflammatory response of the LQ population contrasted with that of the TZ population, showing a notable downregulation in the AA pathway in the TZ population. This finding implies a potential signaling pathway that may contribute to the mitigation of liver inflammation within our TZ population after treatment (Fig. 3, E1, and E2).

### TZ population responses to SC treatment through oxytocin signaling pathway-associated genes

**Fig. 4:**
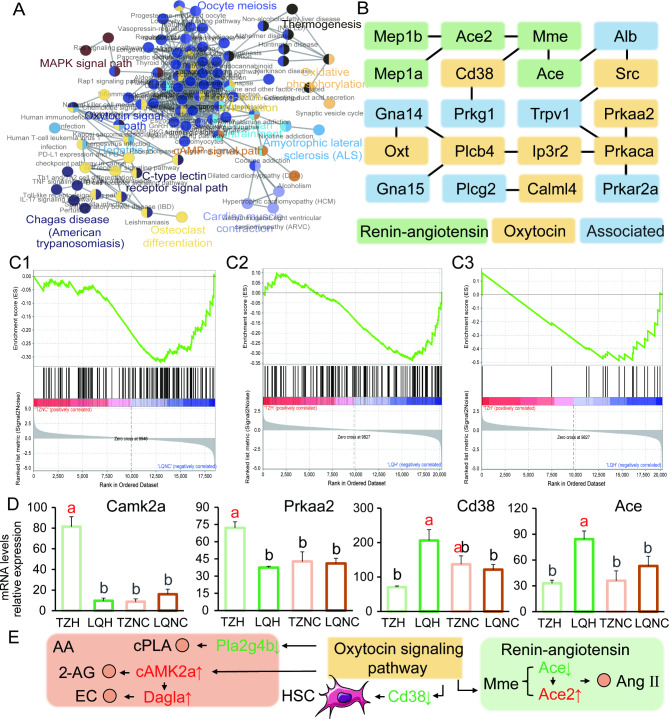
Gene interaction network and pathway analysis of metabolic differences in TZH and LQH hepatic cells. (**A**) Gene interaction network and pathway analysis of metabolic differences in TZ hepatic cells. (**B**) Gene association and interaction in the TZ population after SC treatment in the oxytocin pathway. Boxes represent the genes involved in hepatic metabolism and the line segments represent related pathway correlations. Yellow, green, and blue represent pathways related to oxytocin pathways, renin-angiotensin pathways, and associated genes, respectively. (**C1** and **C2**) GSEA analysis of gene sets for TZ (top) and LQ (bottom) populations before and after SC treatment for oxytocin pathway development and (**C3**) renin-angiotensin pathway development. Positive and negative ES values indicate higher and lower expressions in the TZ population, respectively. Blue and red are low and high levels, respectively. (**D**) Expression profile key genes of the renin-angiotensin and oxytocin pathways in the TZ and LQ populations before and after SC treatment. Letters above the bars indicate significance (ANOVA, *P* < 0.05). TZH, LQH, TZNC, and LQNC are shown in light green, dark green, light yellow, and orange, respectively. (**E**) Key genes and their downstream genes of the renin-angiotensin and AA pathways mediated by oxytocin pathways. Orange circles represent gene-mediated metabolites; green arrows indicate downregulation; red arrows indicate upregulation of genes. 2-AG, 2-arachidonoylglycerol; EC, diacylglycerol lipase-alpha; cPLA, cytosolic phospholipase A2; HSC, hematopoietic stem cells

To identify the unique genes responsible for the mild response of TZ population to liver inflammation, we performed a protein-protein interaction network (PPI) and gene association analysis of the DEGs in the different populations of plateau zokor under the SC treatment. We noticed that DEGs in mitogen-activated protein kinase, oxytocin signaling pathways, and long-term potentiation are associated, which are central pathways in the TZ population that actively regulate detoxification metabolic signaling in the body (Fig. 4A).

DEG enrichment pathways in the TZ population under treatment were all associated with oxytocin signaling. PPI analysis showed that oxytocin DEGs were closely associated with the renin-angiotensin pathway and circadian rhythm as the expression of calcium/calmodulin-dependent protein kinase II Alpha (*Camk2a*) was upregulated by 11-fold, while the protein kinase AMP-activated catalytic subunit alpha 2 (*Prkaa2*), *Cd38*, *Ace*, and calmodulin-like 4 (*Calml4*) expressions were downregulated by 1-, 3-, 3-, and 7-fold, respectively. Additionally, the expressions of Ca + signaling and transcriptional regulators (*Trpv1*) were upregulated 22-fold, while the expression of IP3 receptor isoform 2 (*Ip3r2*) was downregulated 2-fold (Fig. 4B) [see Additional file 2]. In the GSEA analysis, the oxytocin signaling and renin-angiotensin pathway gene sets were upregulated and downregulated after treatment in the TZ population, respectively (Fig. 4, C1, C2, and C3). The *Camk2a* gene mediates the diacylglycerol lipase alpha (*Dagla*) gene co-encoded calcium/calmodulin-dependent protein kinase, a negative regulator of 2-arachidonoylglycerol (2-AG)-mediated synaptic signaling. *Camk2a* produces cannabinoids by hydrolyzing AA, which negatively regulates the AA pathway and accelerates inflammatory clearance in the body (Fig. 4, D and E). This might explain the absence of an obvious inflammatory response in the hepatic tissue of the TZ population after treatment.

### Response of liver detoxification metabolism functions of the TZ population to SC

We identified 122 genes—including UDP glucuronosyltransferase family 1 member A6 (*Ugt1a6a*), *Cyp1a2*, and solute carrier family 27 member 5 (*Slc27a5*), which showed a 3-, 971-, and 1-fold downregulation in their expression, respectively—that were significantly expressed in the hepatic tissue of the TZH group (*P* < 0.05). These genes are involved in the phase I reaction of drug metabolism, oxidative stress, long-term potentiation, inflammatory initiation/differentiation, cell adhesion molecules, chemical carcinogenesis-DNA adducts/receptors, and apoptosis pathways. Thirty-three genes—including *Ugt2b36*, adenylosuccinate synthase 1 (*Adssl1*), and phospholipase C beta 4 (*Plcb4*), which showed a 3-, 23-, and 9-fold downregulation in their expression, respectively—were involved in the combination process of the phase II reaction of drug metabolism and its downstream processes in vivo. Histidine metabolism and primary bile acid biosynthesis, amino acid binding and metabolism, and renin-angiotensin system pathways were significantly expressed in the hepatic tissue of the TZH group (*P* < 0.05). Next, we identified 27 genes—including *Cyp1a2*, glutathione S-transferase mu 5 (*Gstm5*), dihydrodiol dehydrogenase (*Dhdh*), *Gstt4, Ugt2b36*, and microsomal glutathione S-transferase 1 (*Mgst1*), whose expressions were upregulated 4-fold, upregulated 4-fold, downregulated 14-fold, and upregulated 2-fold, respectively—that have been validated with hepatic cytochrome P450 metabolic pathways for detoxification of xenobiotics [see Additional file 2 and 4]. Overall, our findings indicate that the mild response to liver inflammation in the TZ population was related to the binding mechanism of phase II drug metabolism reaction, as well as the reduction in reactive oxygen species (ROS) accumulation. This reduction in ROS accumulation may facilitate the in vivo metabolism of the phase I reaction intermediate, thereby preventing secondary tissue damage (Fig. 2) [33].

### The liver metabolism of DAS events in plateau zokors

The AS analysis was employed to produce distinct mRNA splicing isomers. We found no significant difference in AS event types between the different populations of plateau zokor after the SC treatment. Skipped exon (SE) events in the liver of the TZ population were the main mode of splicing [see Additional file 1 and 5]. However, DAS events played a non-redundant role in response to SC. We found that different mutually exclusive exon (MXE) splicing events increased in the TZ population after treatment [see Additional file 5], suggesting that TZ population respond to SC effects by regulating the exon-skipping form after treatment.

### DAS events and DEGs in the TZ populations adapt to SC in a common and complementary manner

**Fig. 5:**
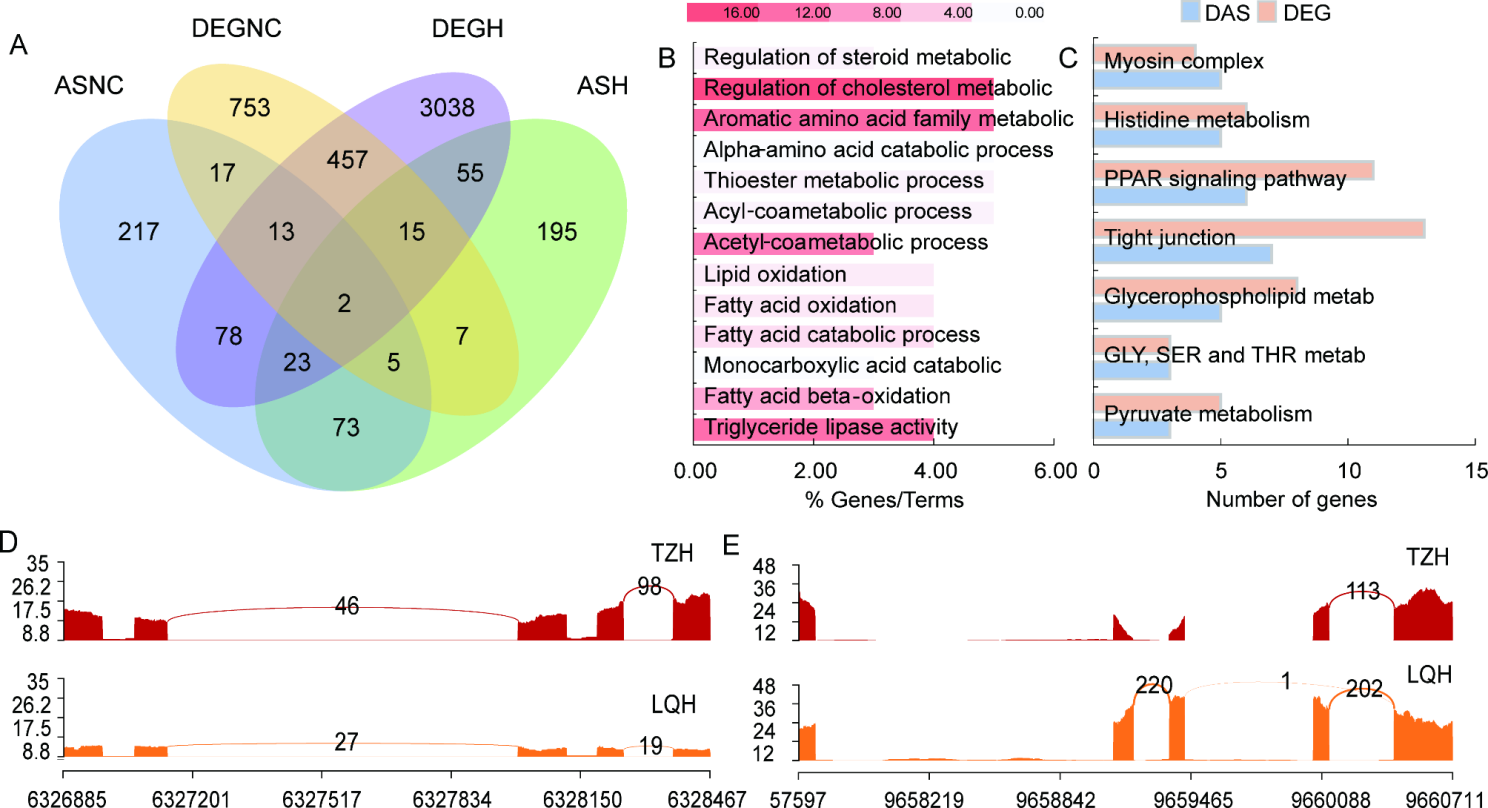
Splicing and connectivity of DAS events and DEGs. (**A**) Venn diagrams showing the gene number of DAS event (by analysis) and DEG overlap. DASNC, differential alternative splicing in the TZ and LQ populations before SC treatment; DEGNC, differentially expressed genes in the TZ and LQ populations before SC treatment; DASH, differential alternative splicing in the TZ and LQ populations after SC treatment; DEGNC, differentially expressed genes in the TZ and LQ populations after SC treatment. (**B**) Coincidental GO function enrichment in DASH and DEGH. Color depth represents the number of genes that are enriched. (**C**) The number of overlapping KEGG pathways of DEGH and DASH. DAS, differential alternative splicing. (**D** and **E**) Sashimi graphs highlighting patterns of AS across all DAS events and DEGs for intron clusters associated with D, *Ccnd1* and F, *Pla2g4b*

We compared the molecular function annotations of genes with DAS events and DEGs and found a small number of gene overlaps in response to SC (Fig. 5A). The GO functions with DEGs and DAS events were co-enriched for amino acid and fatty acid catabolic processes (Fig. 5B) [see Additional file 3 and 6]. This indicated that the TZ population not only had an enhanced ability to metabolize inflammation after treatment, but also changed the diversity of hepatic inflammation-related functions in response (Fig. 5C) [see Additional file 4 and 6]. This may be another reason why the inflammatory response of the TZ population was less pronounced.

After the SC treatment, we found that most of the differential genes in oxytocin, arachidonic acid, and renin-angiotensin pathways participated in mutually exclusive exons (MEX) events in the TZ population. Additionally, AS events were identified, along with a considerable 6-fold upregulation in the expression of the cyclin D *(Ccnd1)* gene. This might have resulted in the enhancement of prostaglandin signaling and the increase in the production of inflammatory mediators (Fig. 5D) [see Additional file 1]. Decreased *Pla2g4b* expression may result in impaired cortical integrity and upregulated permeability, and Pla2g4b splice variants are pathogenic (Fig. 5E) [see Additional file 1]. This may be because hepatic DEGs and DAS events in the TZ population counteract the inflammatory response induced by SC in a common and complementary manner.

## Discussion

Here, we investigated liver differences in plateau zokors between two geographic populations after SC treatment at the physiological, biochemical, and genetic levels. Compared to the LQ population, we found that the TZ population did not exhibit considerable liver damage after the SC treatment. The TZ population demonstrated lower levels of transaminase and liver inflammation responses after treatment. Furthermore, we observed a substantial downregulation in the expression of genes associated with liver inflammation, while noting the activation of oxytocin-related pathways. These findings provide new insights into how herbivores adapt to environmental changes.

### Liver function differences among different plateau zokor populations

Our findings suggest that the TZ population exhibited a lesser hepatic inflammatory response after the SC treatment. These results imply that the different populations of plateau zokors possess distinct detoxification metabolic modes in response to the treatment. This pathway is advantageous for the TZ population because it allows them to maintain a high adaptive capacity despite long-term grassland degradation and simultaneously prevents the serious inflammation effects toxic plants may induce [34], which is consistent with the detox restriction hypothesis [8]. This difference in hepatic function indicates that the liver of plateau zokors in grasslands with different long-term degrees of degradation, adapt differently to the PSMs of SC.

However, physiological changes that reduce immune stress responses in the presence of toxic plants can be dangerous as they may reduce the immunity of the plateau zokors to parasitic and bacterial infections. We found that the genes upregulated in the TZ population were enriched in the oxidation-reduction biological process. This may be because the TZ population retains its own immune function by reducing oxidative stress induced by the PSMs in SC [34]. The enrichment of downregulated genes in the AMPK pathway can manage the upregulated inflammatory pathways [35]. These results suggest that enhancing inflammatory pathways can maintain body function by enhancing immune capacity while reducing immune stress and autophagy after ingestion of toxic PSMs [36]. This further supports our hypothesis that TZ populations maintain an adaptive advantage in the PSMs in SC during environment changes by modulating hepatic immune function and oxidative stress capacity.

### Different populations of plateau zokors adapt to different mechanisms in response to SC

Hepatic inflammation was considerably higher in the LQ population than that in the TZ population after SC treatment. However, in the TZ population, transaminase levels did not increase significantly, indicating that the hepatic tissue of the TZ population was not seriously damaged after the SC treatment. This is broadly consistent with observations in insects and mammals where liver response mechanisms, functional changes, metabolic intensity, and oxidative stress capacity have been shown to respond to adaptations to PSMs [12, 37]. These different adaptive mechanisms represent two potential strategies for the adaptive capacity of herbivores to ingest toxic PSMs in various feeding environments.

### LQ population regulates genes related to the hepatic inflammatory mechanism in response to the SC treatment

Here, the hepatic inflammatory factor after treatment in the LQ population was 2.5-fold higher compared with that of the TZ population, and their livers exhibited an obvious hemorrhagic response (Fig. 1A). This suggests that the hepatic inflammatory response in LQ population is still the main mechanism of detoxification and metabolism under SC treatment in the different populations, in line with the findings of previous studies [32]. After treatment, the LQ population responded by regulating typical detoxification metabolism genes—*Cyp2e1* and *Ampd3—*to improve metabolic processes, the phase I process of P450 hepatic drug metabolism, immune stress, and other related pathways [38]. Among them, genes such as epoxide hydrolase 1 (*Ephx1*) and cell division cycle 25 A (*Cdc25a*)—related to the cell cycle and chemical carcinogenesis-related pathways—were also enriched in the LQ population in response to treatment [39]. The activation of these genes is a key pathway leading to the DNA damage response and an important node for maintaining gene stability. *Ccnd1* was incorrectly spliced in many cancer types [40]. Amplification, AS, and overexpression of this gene alters cell cycle progression and is an important way in which the liver regulates homeostasis. Myosin heavy chain 7 (*Myh7*) and inositol polyphosphate-5-phosphatase D (*Inpp5d*) genes are directly related to immune stress formation, which can regulate cytokine signal transduction in the immune system and metabolism and play an important role in inflammatory processes [41]. The Renin–angiotensin system pathway mediated by *Ace* and *Ace2* in LQ population may also cause inflammatory reaction, which may be one of the reasons for inflammatory reaction in LQ population [42]. The inflammatory response in the hepatic system, as seen in the LQ population, can help the body quickly mount a physiological response to local damage. However, this may increase metabolic and nutrient intake costs and cause liver damage. This mechanism could be a burden for herbivores to survive in the presence of toxic plants.

### TZ population reduces liver damage under SC treatment by regulating the oxytocin pathway

We found that the response to the SC treatment in TZ population was associated with hepatic energy conversion efficiency (amino acid and lipid metabolism), mainly through gene expression of fatty acid desaturase 1 (*Fads1*), hydroxysteroid 17-beta dehydrogenase 4 (*Hsd17b4*), *Cyp2e1*, and other genes, as well as increasing MEX events. *Ccnd1* related to lipid metabolism underwent an exon excision event in the TZ population. The AS event of this gene will alter the functional binding sites of hepatic clearance metabolism and enzymatic activity [36]. Fatty acid metabolism has been shown to regulate the production of mitochondria-promoting ROS in macrophages, whose activation contributes to metabolic rate changes [43]. The upregulated expression of genes enriched in the fatty acid metabolism pathway can cause abnormal hepatic function and inflammation, which is an important regulatory pathway in the AA pathway [44]. However, the TZ population may have bypassed the inflammation pathway to reduce its effects on the liver. They can regulate *Cd38, Pla2g4b*, and other genes through the association of *Camk2a* and *Prkaa2* genes which regulate the oxytocin signaling pathway. Research has found that cPLA regulated by the *Pla2g4b* gene can control the release of inflammatory factor AA, and the AS event of the *Pla2g4b* gene can also affect cell proliferation and survival [45]. This change in the *Pla2g4b* gene can increase the diversity of hepatic metabolic functions by reducing the inflammatory response [46]. This reduces the AA pathway and phase I process of P450 hepatic drug metabolism responses and enhances the hepatic drug metabolism phase II process response and Th1 7/1/2 cell differentiation pathway [47–49]. Our findings indicate that the *Pla2g4b* gene in the TZ population exhibited not only an up-regulation of its expression, but also the occurrence of exon skipping events. This result is consistent with the detoxification limitation hypothesis that the molecular costs and benefits associated with different foraging strategies differ [18].

### The phase II process of liver drug metabolism may help the TZ population respond to SC effects

Our previous study found that the metabolism of cytochrome P450 enzymes to xenobiotics in vivo increased in the LQ population during the SC treatment [32]. Although this process can metabolize drugs/compounds into hydrophilic substances, it can also produce the accumulation of many ROS, and the generated hydrophilic compounds may be more toxic. At this time, if the phase II process of liver drug metabolism cannot be accelerated, the liver will be damaged by ROS [50]. After receiving SC treatment, the SLC gene family and UGT gene family, which are related to the phase II process of liver drug metabolism, were significantly up-regulated in the TZ population. This increase in substrate transport and reduction in ROS can inhibit the tissue damage caused by the phase I process of P450 hepatic drug metabolism [51]. The diterpenoids generated during this process can resist oxidative stress damage. The result of this phenomenon is that the TZ population might potentially reduce secondary damage to cells caused by the intermediate metabolites produced in the body during the phase II process of P450 hepatic drug metabolism.

### The impact of increased adaptive capacity in adaptation to toxic plants

The arms race hypothesis between plants and herbivores posits that populations that have long survived in the presence of toxic plants have a higher and more complex detoxification metabolism than those of other populations. Owing to the environment of the TZ population where toxic plant invasion is more severe, they can maintain a diverse and high-level adaptability to toxic PSMs in vivo allowing them to improve their survival rates in areas with environmental changes. Therefore, the TZ population may have an advantage over the LQ population in normal vegetation structures in their ability to adapt to environmental changes caused by climate change and anthropogenic activities. However, the limitation of this study is that it only focused on the examination of gene expression pertaining to detoxification metabolism. Subsequently, we aim to elucidate the adaptability of herbivores towards poisonous plants through correlation analyses between metabolism and gut microbes.

## Conclusions

Our work supports the conclusion that herbivores have extensive toxic plant adaptations through mechanisms of hepatic function regulation; however, the current understanding of this relationship remains incomplete. Further research is required on differential metabolites and the underlying mechanisms by which microorganisms coordinate and regulate adaptive capacity. This will provide a reference for understanding the impact of environmental changes on species and their coping strategies.

## Materials and methods

### Capture, husbandry, and SC collection

The Tianzhu (TZ) population of the plateau zokor lives close to Tianzhu city, where grazing pressure and the degree of interference from human activities are high, the grassland is severely degraded, and toxic weeds are widespread. *Stellera chamaejasme* (SC) is currently the dominant plant in the TZ grassland and is consumed by the local plateau zokors [see Additional file 1] [27, 52]. Twenty adult plateau zokors (223.4 ± 47.9 g) were captured from their habitat in the TZ County grasslands in the northwest part of the QTP (37.114526°N, 103.171024°E), Gansu Province, China. The Luqu (LQ) population live in the Luqu area where the grassland has less grazing pressure, the area is richer in vegetation with a wide range of food, and there are fewer SC plants than in the TZ grasslands [see Additional file 1]. The same capture method was used to obtain 20 adult plateau zokors (235.9 ± 56.9 g) in the LQ area (34.258061°N, 102.367445°E). Specialized noninvasive tube traps (Baoji Ludixincheng Co Ltd Xi’an China) were used to capture the plateau zokor in their TZ and LQ habitats from September to October 2020. Once captured, they were taken to a local laboratory and examined for damage and disease. Efforts were made to minimize the metabolic disparities in the liver induced by injuries and diseases to the greatest extent feasible. Upon arrival, the animals were placed in 37 × 70 × 37 cm plastic containers, and soil from the plateau zokor habitat was used as nesting material to minimize their stress. The animals were housed in the laboratory for seven days without contact to reduce stress and given ad libitum access to their preferred food (carrots and potatoes) during the acclimatization and experimental period. The temperature was maintained at 12 ± 4 °C and humidity at 45–55% [53]. Our experiments were approved by the Animal Ethics Committee of Gansu Agricultural University and supported by the local government (GAU-LC-2020-014).

The material and structure of the prepared SC are described separately [see Additional file 7 and 8]. Fresh SC plants were collected, placed in paper bags, sealed, and preserved. Identification was done by Professor Su Junhu of the Grassland College of Gansu Agricultural University, and the specimen was stored in the herbarium of the same university. Fresh SC roots were dried and crushed into a powder with a mass of 56.73 g/100 g. The powder was soaked with distilled water, boiled, and filtered twice. The filtrate was then concentrated to obtain the SC root concentrate, and kept at a volume of 5.3 g/mL for further analysis [54].

### Experimental plan

After one week of the acclimation period, the animals from TZ and LQ were randomly divided into two groups of 10 zokors each (five males and five females). Based on previously determined standards, the doses in each group were as follows: TZ normal control (TZNC), LQ normal control (LQNC), TZ high dose (TZH: 2.1 mL/kg), and LQ high dose (LQH: 2.1 mL/kg). Throughout the experimental period, each group was treated between 8 and 10 am. Administered by oral gavage once daily for 7 d, the control and treated groups were given 0.1 mL/100 g body weight of distilled water and SC aqueous extract, respectively. Subsequently, all animals were euthanized humanely, and their livers and plasma removed and stored until further analysis. Biochemical levels were determined using serum isolated from the plasma. Liver samples were preserved by fixing them in 10% neutral formalin and immediately placing them in liquid nitrogen for transcriptomic evaluation.

### Hepatic pathology tests

Liver tissue was removed after being fixed for 1 d. Two pieces, each 0.5 cm^3^, were removed from the center and beginning margins of the liver and embedded in paraffin wax. The embedded wax blocks were cut into 5 μm-thick sections using a Leica rotary sectioning machine (Leica RM2265), and the tissue was stained with hematoxylin and eosin. The extent of hepatic damage and inflammation in the tissues was observed under a light microscope (Panthera U, Motic®, Xiamen, China). The number of inflammatory cells was calculated using Image Pro Plus software (version 6.0). The number of inflammatory cells in the liver and the level of inflammation were compared using H_2_O.

### Determination of oxidative stress and transaminase activity

Serum biochemical indicators—aspartate aminotransferase (AST), alanine aminotransferase (ALT), and malondialdehyde (MDA)—were determined according to the instructions of the Nanjing Jiancheng Institute of Biotechnology (Nanjing, Jiangsu, China). Briefly, the prepared serum was subjected to a reaction with 2,4-dinitrophenylhydrazine at 37 °C for 20 min to determine the transaminase level. The MDA concentration in the serum was assessed by its reaction with thiobarbituric acid, resulting in the formation of a pink chromogen compound. To determine biochemical levels using a spectrophotometer (Biotek Epoch 2), the transaminase optical density was set to 510 nm, and the values expressed in U/L. The MDA assay optical density was set to 532 nm and the values expressed in mmol/L. The AST, ALT, and MDA levels of all samples under investigation were measured by introducing H_2_O into three wells as controls.

### Transcriptome sequencing and gene function analysis

We carefully chose three samples exhibiting distinct alterations in liver inflammation without any other problems from the LQH group. Additionally, we selected three distinct samples of liver cells from each of the remaining three groups (TZH = 3, LQNC = 3, TZNC = 3) for transcriptome sequencing. RNA extraction, purification, cDNA library construction, transcript assembly, and gene function annotation are described separately [see Additional file 7].

### Statistical analysis

Data visualization of inflammatory cell counts and mRNA expression were presented using Microsoft PowerPoint software (version 16.0.15629.20156). To establish the exclusive influence of SC on the outcomes, genes exhibiting consistent differential expression patterns before and after SC treatment in different populations of plateau zokors were excluded from the analysis. We used SPSS statistical software (version 19.0) to analyze biochemical indicators and gene expressions, expressed as mean ± SE (n = 3). Means were compared using ANOVA, and statistical significance was set at *P* < 0.05. Duncan’s post-hoc test was used after the one-way ANOVA. AS and differential alternative splicing (DAS) event gene functions and pathways were enrichment analyzed using Cytoscape (version 3.9.0).

### Electronic supplementary material

Below is the link to the electronic supplementary material.

**Figure S1**. The study area and information on the plateau zokors and *Stellera chamaejasme*. Locations of sampling points of Tianzhu (TZ) and Luqu (LQ) populations (A). Details of *Stellera chamaejasme* biology and its secondary plant metabolites (PSMs) (B). LQ population and an image of their habitat (C1). TZ population and image of their habitat (C2). **Figure S2**. Differentially expressed genes (DEGs) and gene function enrichment analysis of plateau zokor liver in response to SC treatment. Homologous differential expression consistency between the significantly differentially expressed genes of the TZ and LQ plateau zokor populations before and after SC treatment, where each dot represents one gene. Transcripts were considered differentially expressed at *P*-value < 0.05 and |Log2FoldChange| > 1, with significant upregulation and downregulation shown in red and blue, respectively (A and C). Comparison of KEGG pathways of DEGs in the TZ and LQ populations before and after SC treatment. Color depth represents how many genes are enriched. (B and D). Comparison of the GO term of genes in the TZ and LQ populations (E1) before and (F1) after SC treatment. Comparison of the GO function proportions of the DEGs in the TZ and LQ populations (E2) before and (F2) after SC treatment. MF, molecular function; BP, biological process; CC, cellular component. (G) Red represents the top ten GO functions upregulated by TZ compared with the LQ population; green represents the top ten GO functions downregulated by TZ compared with the LQ population. (H) Red represents the top ten KEGG pathways upregulated by TZ compared with the LQ population; green represents the top ten KEGG pathways downregulated by TZ compared with the LQ population. **Figure S3**. GO functional enrichment. Comparison of the GO function of genes in the TZ and LQ populations before and after SC treatment. (A) Top 30 GO functions upregulated by TZ compared with the LQ population after SC treatment. (B) Top 30 GO functions downregulated by TZ compared with the LQ population after SC treatment. (C) Top 30 GO functions DEGs by TZ compared with the LQ population after SC treatment. (D) Top 30 GO functions DEGs by TZ compared with the LQ population before SC treatment. (E) Top 30 GO functions downregulated by TZ compared with the LQ population before SC treatment. (F) Top 30 GO functions upregulated by TZ compared with the LQ population before SC treatment. **Figure S4**. KEGG enrichment pathway. Comparison of the KEGG function of genes in the TZ and LQ populations before and after SC treatment. (A) Top 30 KEGG functions downregulated by TZ compared with the LQ population after SC treatment. (B) Top 30 KEGG functions upregulated by TZ compared with the LQ population after SC treatment. (C) Top 30 KEGG functions DEGs by TZ compared with the LQ population after SC treatment. (D) Top 30 KEGG functions DEGs by TZ compared with the LQ population before SC treatment. (E) Top 30 KEGG functions downregulated by TZ compared with the LQ population before SC treatment. (F) Top 30 KEGG functions upregulated by TZ compared with the LQ population before SC treatment. **Figure S5**. Complex AS events identified between TZ and LQ populations. A1, A2/B1, B2: Distribution of (AS/DAS) identified by comparing TZH and LQH, and TZNC and LQNC. DAS, differential alternative splicing; SE, skipped exons; RI, retained introns; MXE, mutually exclusive exons; A5’SS, alternative 5’ splice sites; A3’SS, alternative 3’ splice sites. C1, C2: Venn diagrams showing the amount of overlap of spliced regions for TZNC/LQNC and TZH/LQH, DAS (by analyses) for TZNC/LQNC and TZH/LQH. D1, D2, D3: The distribution in DAS events was determined. D1: Unique events in TZH and LQH. D2: Coincidental events in TZNC/LQNC and TZH/LQH. D3: Unique events in TZNC and LQNC. E1/E2: DAS KEGG terms in TZNC and LQNC/TZH and LQH. **Figure S6**. *Ccnd1* and *Pla2g4b* sashimi graphs patterns of AS. (A) Sashimi graphs highlighting patterns of *Ccnd1* DAS after SC treatment. (B) Sashimi graphs highlighting patterns of *Pla2g4b* DAS after SC treatment. The arcs represent splice-junction-connected exons. *Ccnd1*, Cyclin D1; *Pla2g4b*, phospholipase A2 group IVB.

**Additional file 2**: S1 TZHvsLQH Differentially expressed genes (DEGs). S2 TZNCvsLQNC Differentially expressed genes (DEGs). S3 TZHvsLQH Differentially expressed downregulated genes (DEGs). S4 TZHvsLQH Differentially expressed upregulated genes (DEGs). S5 TZNCvsLQNC Differentially expressed downregulated genes (DEGs). S6 TZNCvsLQNC Differentially expressed upregulated genes (DEGs).

**Additional file 3**: S1 TZNC vs LQNC GO functions enrichment analysis. S2 TZNC vs LQNC downregulated GO functions enrichment analysis. S3 TZNC vs LQNC upregulated GO functions enrichment analysis. S4 TZH vs LQH GO functions enrichment analysis. S5 TZH vs LQH downregulated GO functions enrichment analysis. S6 TZH vs LQH upregulated GO functions enrichment analysis.

**Additional file 4**: S1 TZNC vs LQNC KEGG pathways enrichment analysis. S2 TZNC vs LQNC downregulated KEGG pathways enrichment analysis. S3 TZNC vs LQNC upregulated KEGG pathways enrichment analysis. S4 TZH vs LQH KEGG pathways enrichment analysis. S5 TZH vs LQH downregulated KEGG pathways enrichment analysis. S6 TZH vs LQH upregulated KEGG pathways enrichment analysis.

**Additional file 5**: S1 Distribution of skipped exons identified by comparing TZNC and LQNC. S2 Distribution of retained introns identified by comparing TZNC and LQNC. S3 Distribution of mutually exclusive exons identified by comparing TZNC and LQNC. S4 Distribution of alternative 3’ splice sites identified by comparing TZNC and LQNC. S5 Distribution of alternative 5’ splice sites identified by comparing TZNC and LQNC. S6 Distribution of skipped exons identified by comparing TZH and LQH. S7 Distribution of retained introns identified by comparing TZH and LQH. S8 Distribution of mutually exclusive exons identified by comparing TZH and LQH. S9 Distribution of alternative 3’ splice sites identified by comparing TZH and LQH. S10 Distribution of alternative 5’ splice sites identified by comparing TZH and LQH. S11 Distribution of skipped exons identified by comparing TZNC and LQNC. S12 Distribution of retained introns identified by comparing TZNC and LQNC. S13 Distribution of mutually exclusive exons identified by comparing TZNC and LQNC. S14 Distribution of alternative 3’ splice sites identified by comparing TZNC and LQNC. S15 Distribution of alternative 5’ splice sites identified by comparing TZNC and LQNC. S16 Distribution of alternative 3’ splice sites identified by comparing TZH and LQH. S17 Distribution of skipped exons identified by comparing TZH and LQH. S18 Distribution of retained introns identified by comparing TZH and LQH. S19 Distribution of mutually exclusive exons identified by comparing TZH and LQH. S20 Distribution of alternative 5’ splice sites identified by comparing TZH and LQH.

**Additional file 6**: S1 Comparison of Immune System Process of DAS in the TZ and LQ populations before SC treatment. S2 Comparison of Molecular Function of DAS in the TZ and LQ populations before SC treatment. S3 Comparison of Cellular Component of DAS in the TZ and LQ populations before SC treatment. S4 Comparison of Biological Process of DAS in the TZ and LQ populations before SC treatment. S5 Comparison of KEGG pathways of DAS in the TZ and LQ populations before SC treatment. S6 Comparison of Immune System Process of DAS in the TZ and LQ populations after SC treatment. S7 Comparison of Molecular Function of DAS in the TZ and LQ populations after SC treatment. S8 Comparison of Cellular Component of DAS in the TZ and LQ populations after SC treatment. S9 Comparison of Biological Process of DAS in the TZ and LQ populations after SC treatment. S10 Comparison of KEGG pathways of DAS in the TZ and LQ populations after SC treatment.

Supplementary Material 7

**Additional file 8**: Information about compounds supposedly identified in *Stellera chamaejasme.*

## Data Availability

We deposited all animals as voucher specimens in Gansu Agricultural University. All sequencing data (both sequencing reads and the transcriptome assembly) will be made available through Genbank (NCBI), BioProject number: PRJNA770149, PRJNA910669.

## Abbreviations

PSMs: Plant secondary metabolites
2-AG: 2-arachidonoylglycerol
AA: Arachidonic acid
Ace: Angiotensin I Converting Enzyme
Adssl1: Adenylosuccinate synthase 1
ALT: Alanine aminotransferase
Ampd3: Adenosine monophosphate deaminase 3
AMPK α2: AMP-activated catalytic subunit α2
Ang II: Angiotensin II
AS: Alternative splicing
AST: Aspartate aminotransferase
CACN: Calcium voltage-gated channel auxiliary subunit alpha 2 delta 2
Calml4: Calmodulin like 4
Camk2a: Calcium/Calmodulin Dependent Protein Kinase II Alpha
Ccnd1: Cyclin D
Cd38: CD38 Molecule
Cmtm7: CKLF-like marvel transmembrane domain containing 7
cPLA2: Phospholipase A2
CYP: Cytochrome P450
CYS: Cysteine
Dagla: Diacylglycerol lipase alpha
DEGs: Differentially expressed genes
Dhdh: Dihydrodiol dehydrogenase
Fabp3: Fatty acid binding protein 3
GLU: Glutamic
GLY: Glycine
GO: Gene ontology
GSEA: Gene set enrichment analysis
Gstm5: Glutathione S-transferase mu 5
GSTs: Glutathione S-transferase
Gstt4: Glutathione S-transferase theta 4
Ifitm2: Interferon induced transmembrane protein 2
Ip3r2: IP3 receptor isoform 2
KEGG: Kyoto encyclopedia of genes and genomes
LTs: Leukotrienes
MDA: Malondialdehyde
MEK1/2: Map kinase kinase ½
Mgst1: Microsomal glutathione S-transferase 1
Mme: Membrane metalloendopeptidase
MXE: Mutually exclusive exon
Nudt1: Nudix hydrolase 1
PGs: Prostaglandins
PKA: Protein kinase camp-activated catalytic subunit alpha
Pla2g4b: Phospholipase A2 group IVB
Plcb4: Phospholipase C beta 4
PPI: Protein-protein interaction network
Prkaa2: Protein kinase AMP-activated catalytic subunit alpha 2
RAP1: Ras-related protein 1a
RAS: Hras proto-oncogene
ROS: Reactive oxygen species
RSK2: Ribosomal protein S6 kinase A2
SC: *Stellera chamaejasme*
SE: Skipped exon
Tcfl5: Transcription factor like 5
Trpv1: Transcriptional regulators
Trpv4: Transient receptor potential cation channel subfamily V member 4
TXAs: Thromboxanes
UGT: UDP glycosyltransferase
