# Supplementary material for: Comparative transcriptomes reveal geographic differences in the ability of the liver of plateau zokors (Eospalax baileyi) to respond and adapt to toxic plants
Source: BMC Genomics. 2023 Sep 6;24:529. doi: 10.1186/s12864-023-09642-5 (PMC10483729; doi:10.1186/s12864-023-09642-5)
Supplement: Supplementary file 1 — Figure S1. The study area and information on the plateau zokors and Stellera chamaejasme. Locations of sampling points of Tianzhu (TZ) and Luqu (LQ) populations (A). Details of Stellera chamaejasme biology and its secondary plant metabolites (PSMs) (B). LQ population and an image of their habitat (C1). TZ population and image of their habitat (C2). Figure S2. Differentially expressed genes (DEGs) and gene function enrichment analysis of plateau zokor liver in response to SC treatment. Homologous differential expression consistency between the significantly differentially expressed genes of the TZ and LQ plateau zokor populations before and after SC treatment, where each dot represents one gene. Transcripts were considered differentially expressed at P-value < 0.05 and |Log2FoldChange| > 1, with significant upregulation and downregulation shown in red and blue, respectively (A and C). Comparison of KEGG pathways of DEGs in the TZ and LQ populations before and after SC treatment. Color depth represents how many genes are enriched. (B and D). Comparison of the GO term of genes in the TZ and LQ populations (E1) before and (F1) after SC treatment. Comparison of the GO function proportions of the DEGs in the TZ and LQ populations (E2) before and (F2) after SC treatment. MF, molecular function; BP, biological process; CC, cellular component. (G) Red represents the top ten GO functions upregulated by TZ compared with the LQ population; green represents the top ten GO functions downregulated by TZ compared with the LQ population. (H) Red represents the top ten KEGG pathways upregulated by TZ compared with the LQ population; green represents the top ten KEGG pathways downregulated by TZ compared with the LQ population. Figure S3. GO functional enrichment. Comparison of the GO function of genes in the TZ and LQ populations before and after SC treatment. (A) Top 30 GO functions upregulated by TZ compared with the LQ population after SC treatment. (B) Top 30 GO functions downreg [file 12864_2023_9642_MOESM1_ESM.docx]

**Additional file 1**

**Comparative transcriptomes reveal geographic differences in the ability of the liver of plateau zokors (*Eospalax baileyi*) to respond and adapt to toxic plants**

**Co-Author Details:**

Yuchen Tan^a,b^, Yanli Wang^a,b^, Qianqian Liu^a,b^, Zhicheng Wang^a,b^, Shangli Shi^a,b^, Junhu Su^a,b,*^

^a^ College of Grassland Science, Key Laboratory of Grassland Ecosystem (Ministry of Education), Gansu Agricultural University, Lanzhou 730070, China

^b^ Gansu Agricultural University-Massey University Research Centre for Grassland Biodiversity, Gansu Agricultural University, Lanzhou 730070, China

**^*^ Correspondence Author:**

College of Grassland Science, Key Laboratory of Grassland Ecosystem (Ministry of Education), Gansu Agricultural University, Lanzhou 730070, China

E-mail: sujh@gsau.edu.cn. Tel. +86-931-7631213, Fax: +86-931-7631227.


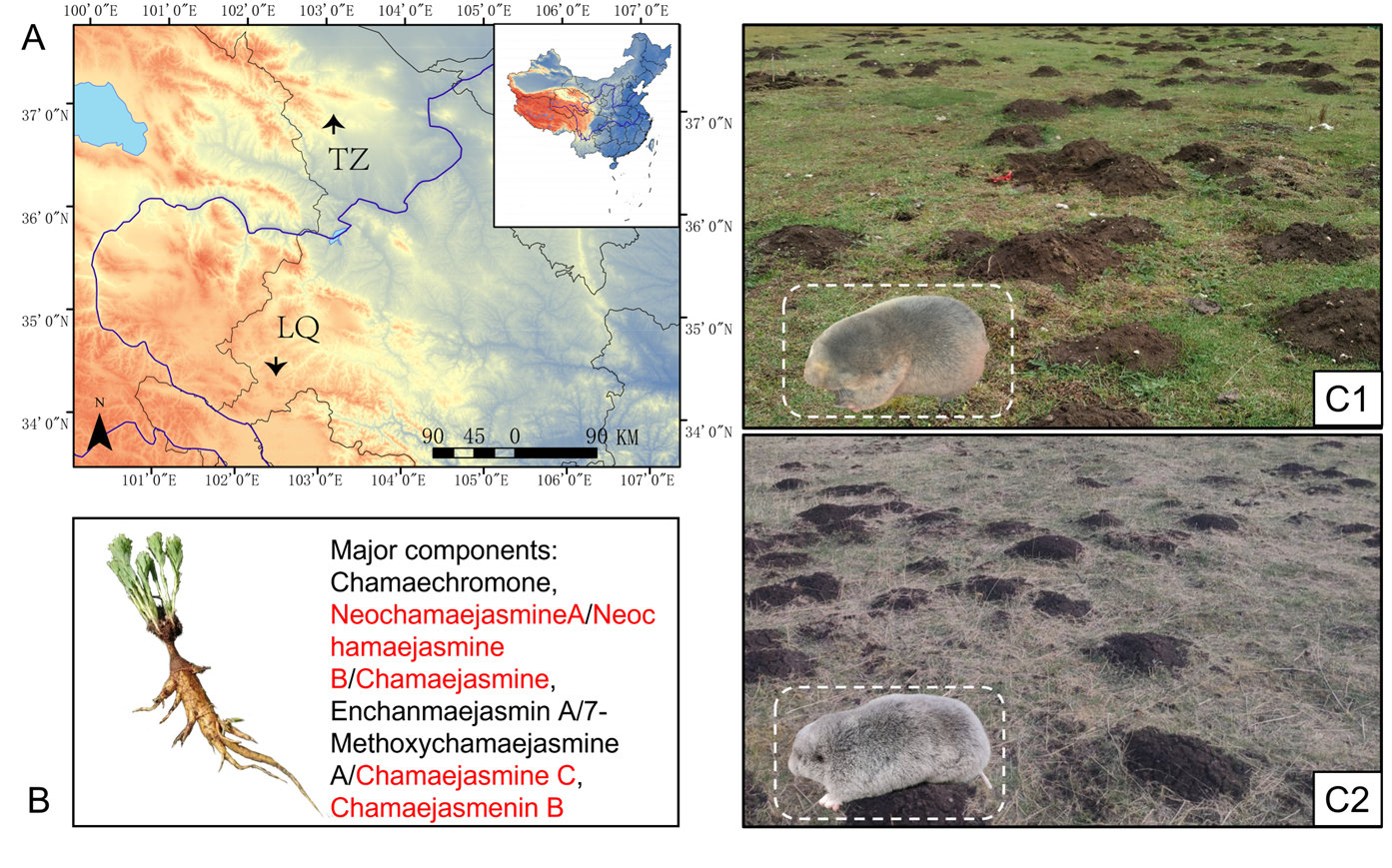


**Figure S1**. The study area and information on the plateau zokors and *Stellera chamaejasme*. Locations of sampling points of Tianzhu (TZ) and Luqu (LQ) populations (A). Details of *Stellera chamaejasme* biology and its secondary plant metabolites (PSMs) (B). LQ population and an image of their habitat (C1). TZ population and image of their habitat (C2).


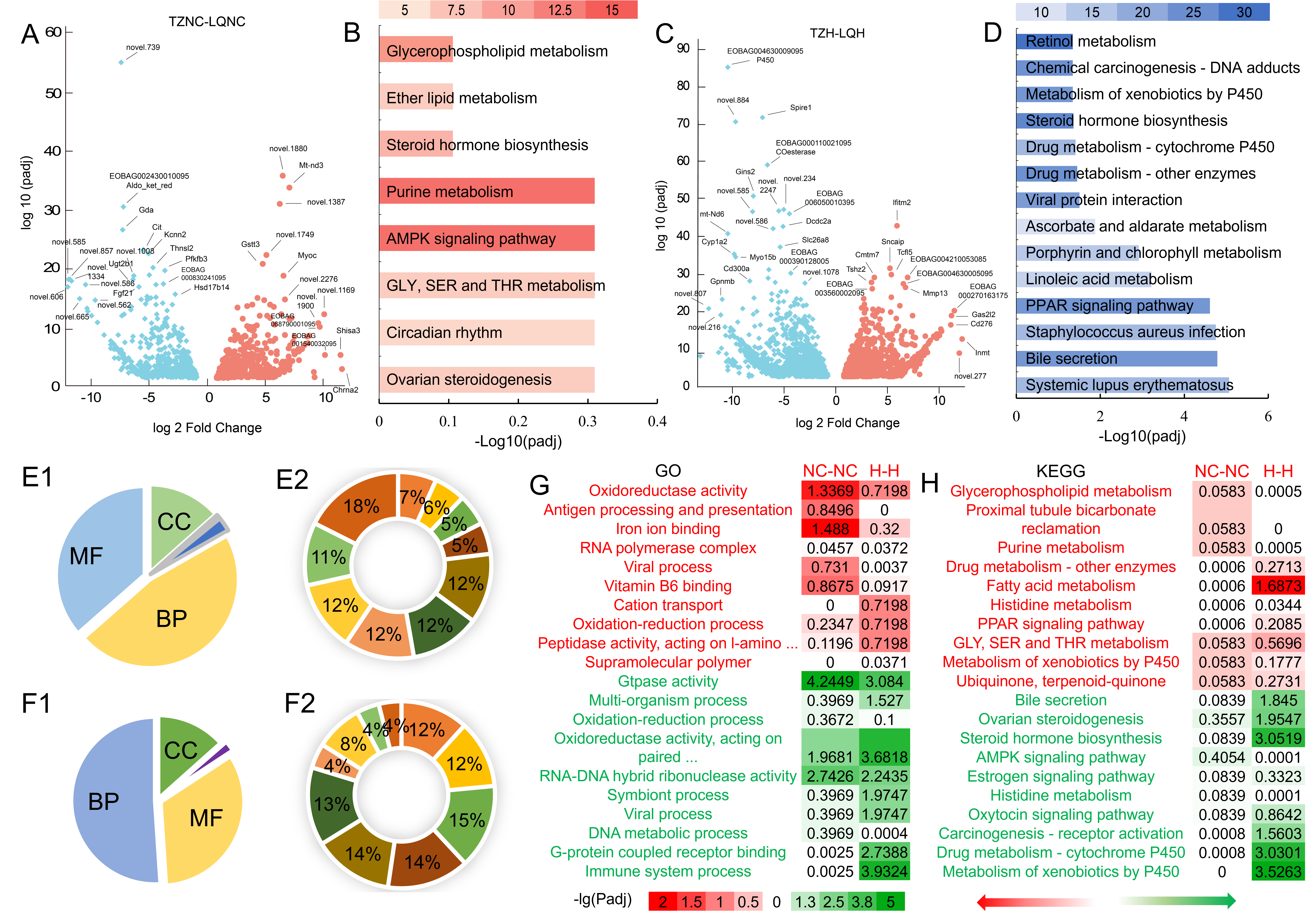


**Figure S2**. Differentially expressed genes (DEGs) and gene function enrichment analysis of plateau zokor liver in response to SC treatment. Homologous differential expression consistency between the significantly differentially expressed genes of the TZ and LQ plateau zokor populations before and after SC treatment, where each dot represents one gene. Transcripts were considered differentially expressed at p-value <0.05 and |Log2FoldChange| > 1, with significant upregulation and downregulation shown in red and blue, respectively (A and C). Comparison of KEGG pathways of DEGs in the TZ and LQ populations before and after SC treatment. Color depth represents how many genes are enriched. (B and D). Comparison of the GO term of genes in the TZ and LQ populations (E1) before and (F1) after SC treatment. Comparison of the GO function proportions of the DEGs in the TZ and LQ populations (E2) before and (F2) after SC treatment. MF, molecular function; BP, biological process; CC, cellular component. (G) Red represents the top ten GO functions upregulated by TZ compared with the LQ population; green represents the top ten GO functions downregulated by TZ compared with the LQ population. (H) Red represents the top ten KEGG pathways upregulated by TZ compared with the LQ population; green represents the top ten KEGG pathways downregulated by TZ compared with the LQ population.

**
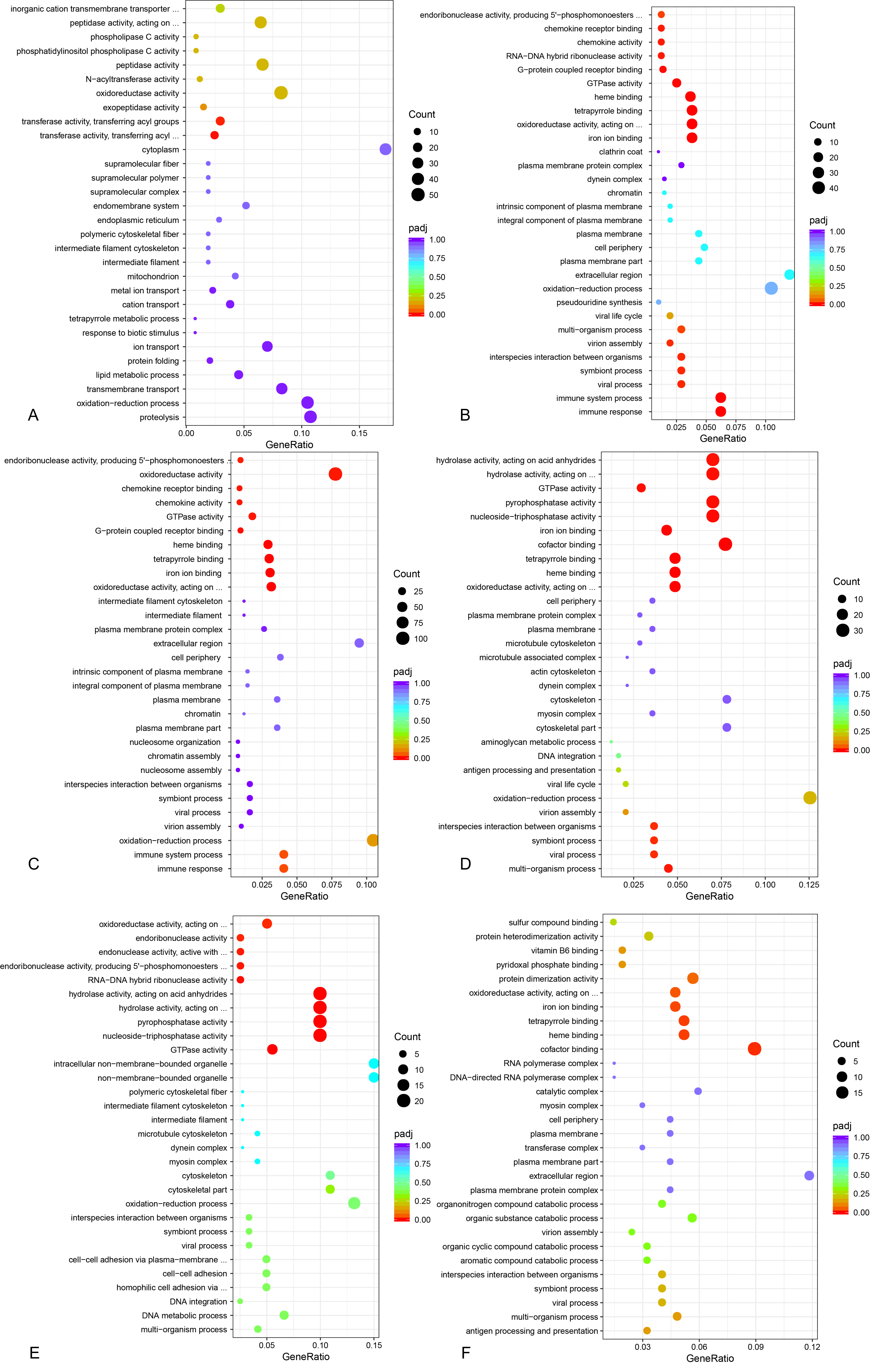
**

**Figure S3.** GO functional enrichment. Comparison of the GO function of genes in the TZ and LQ populations before and after SC treatment. (A) Top 30 GO functions upregulated by TZ compared with the LQ population after SC treatment. (B) Top 30 GO functions downregulated by TZ compared with the LQ population after SC treatment. (C) Top 30 GO functions DEGs by TZ compared with the LQ population after SC treatment. (D) Top 30 GO functions DEGs by TZ compared with the LQ population before SC treatment. (E) Top 30 GO functions downregulated by TZ compared with the LQ population before SC treatment. (F) Top 30 GO functions upregulated by TZ compared with the LQ population before SC treatment.


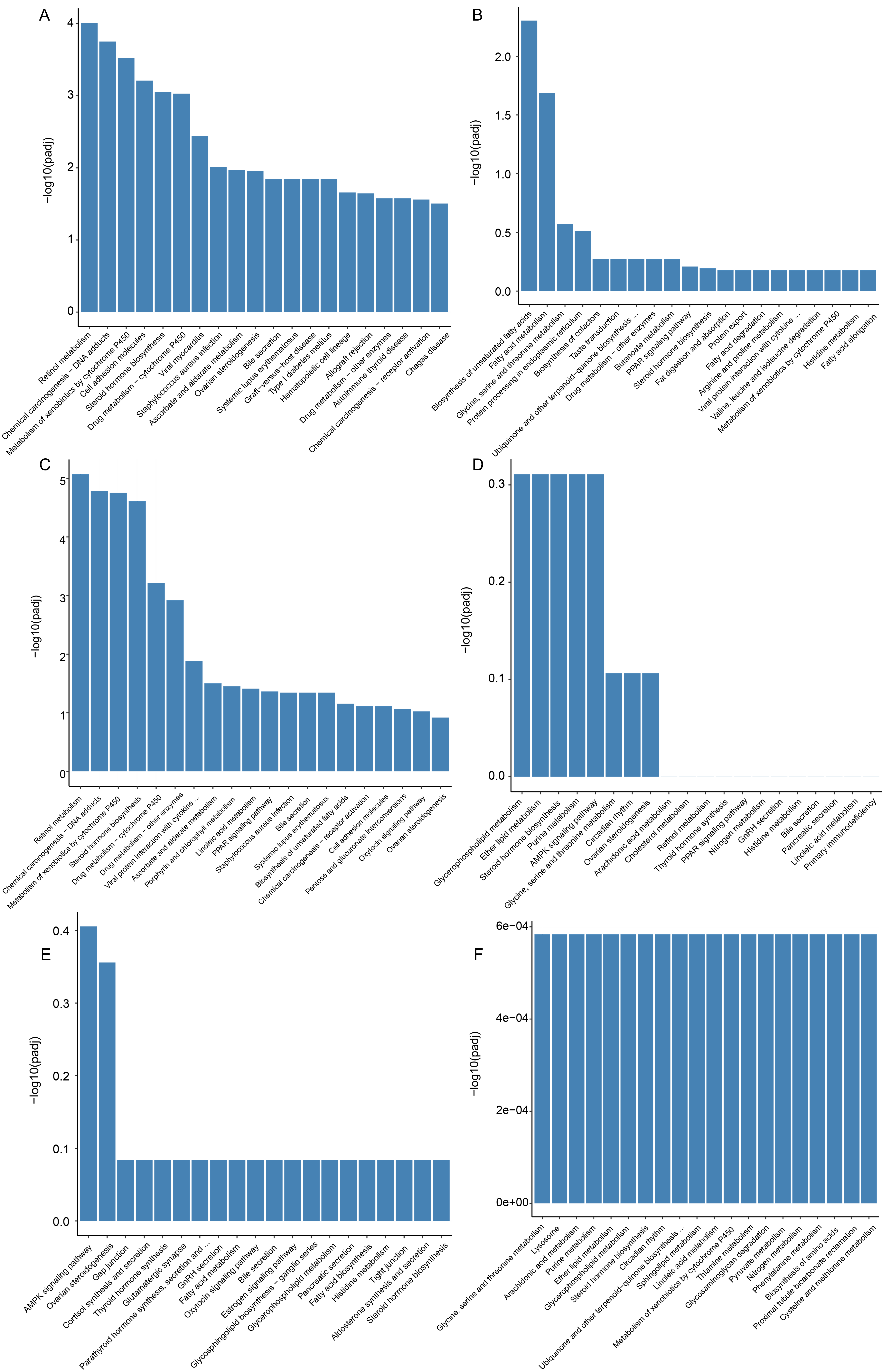


**Figure S4.** KEGG enrichment pathway. Comparison of the KEGG function of genes in the TZ and LQ populations before and after SC treatment. (A) Top 30 KEGG functions downregulated by TZ compared with the LQ population after SC treatment. (B) Top 30 KEGG functions upregulated by TZ compared with the LQ population after SC treatment. (C) Top 30 KEGG functions DEGs by TZ compared with the LQ population after SC treatment. (D) Top 30 KEGG functions DEGs by TZ compared with the LQ population before SC treatment. (E) Top 30 KEGG functions downregulated by TZ compared with the LQ population before SC treatment. (F) Top 30 KEGG functions upregulated by TZ compared with the LQ population before SC treatment.


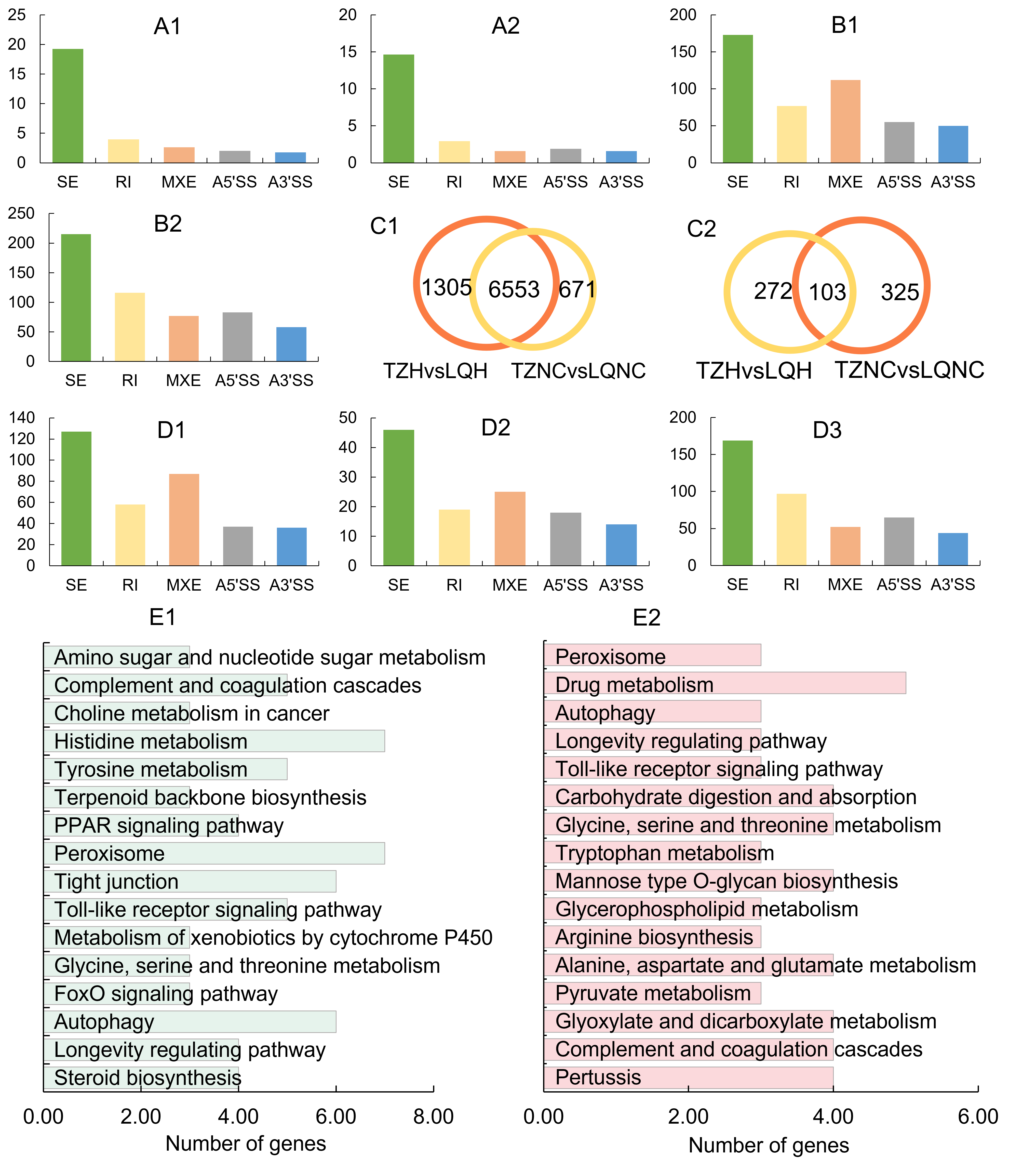


**Figure S5.** Complex AS events identified between TZ and LQ populations. A1, A2/B1, B2: Distribution of (AS/DAS) identified by comparing TZH and LQH, and TZNC and LQNC. DAS, differential alternative splicing; SE, skipped exons; RI, retained introns; MXE, mutually exclusive exons; A5’SS, alternative 5’ splice sites; A3’SS, alternative 3’splice sites. C1, C2: Venn diagrams showing the amount of overlap of spliced regions for TZNC/LQNC and TZH/LQH, DAS (by analyses) for TZNC/LQNC and TZH/LQH. D1, D2, D3: The distribution in DAS events was determined. D1: Unique events in TZH and LQH. D2: Coincidental events in TZNC/LQNC and TZH/LQH. D3: Unique events in TZNC and LQNC. E1/E2: DAS KEGG terms in TZNC and LQNC/TZH and LQH.


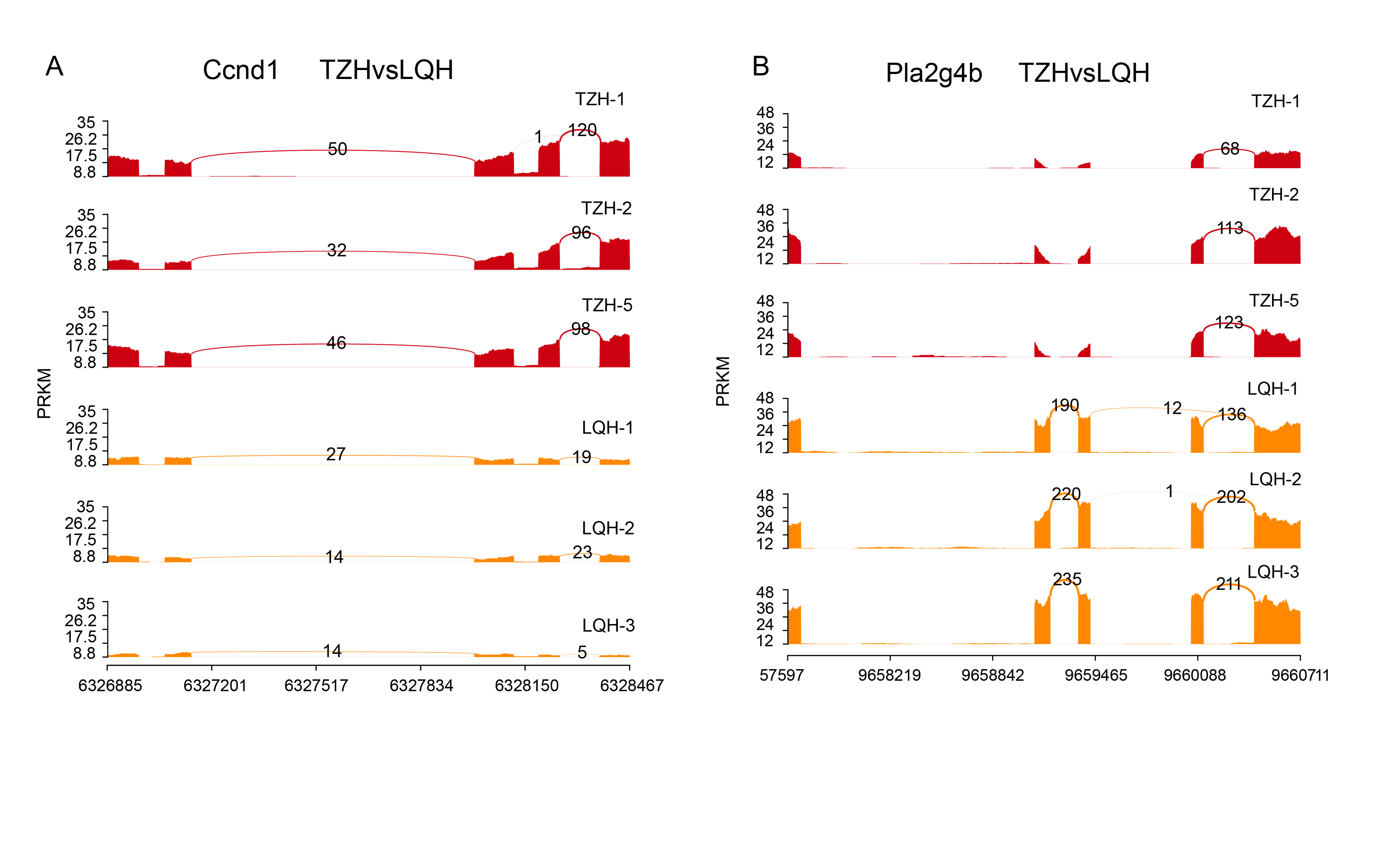


**Figure S6.** *Ccnd1* and *Pla2g4b* sashimi graphs patterns of AS. (A) Sashimi graphs highlighting patterns of *Ccnd1* DAS after SC treatment. (B) Sashimi graphs highlighting patterns of *Pla2g4b* DAS after SC treatment. The arcs represent splice-junction-connected exons. *Ccnd1*, Cyclin D1; *Pla2g4b*, phospholipase A2 group IVB.
