# Supplementary material for: Comparative transcriptomes reveal geographic differences in the ability of the liver of plateau zokors (Eospalax baileyi) to respond and adapt to toxic plants
Source: BMC Genomics. 2023 Sep 6;24:529. doi: 10.1186/s12864-023-09642-5 (PMC10483729; doi:10.1186/s12864-023-09642-5)
Supplement: Supplementary file 7 — Supplementary Material 7 [file 12864_2023_9642_MOESM7_ESM.docx]

**Additional file 7**

**Comparative transcriptomes reveal geographic differences in the ability of the liver of plateau zokors (*Eospalax baileyi*) to respond and adapt to toxic plants**

**Co-Author Details:**

Yuchen Tan^a,b^, Yanli Wang^a,b^, Qianqian Liu^a,b^, Zhicheng Wang^a,b^, Shangli Shi^a,b^, Junhu Su^a,b,*^

^a^ College of Grassland Science, Key Laboratory of Grassland Ecosystem (Ministry of Education), Gansu Agricultural University, Lanzhou 730070, China

^b^ Gansu Agricultural University-Massey University Research Centre for Grassland Biodiversity, Gansu Agricultural University, Lanzhou 730070, China

**^*^ Correspondence Author:**

College of Grassland Science, Key Laboratory of Grassland Ecosystem (Ministry of Education), Gansu Agricultural University, Lanzhou 730070, China

E-mail: sujh@gsau.edu.cn. Tel. +86-931-7631213, Fax: +86-931-7631227.

**Materials and Methods**

**SC collection**

Fresh SC plant material was collected from the Tianzhu plateau zokors’ natural habitat in Tianzhu County in the northwest part of the QTP (37.114526°N, 103.171024°E), Gansu Province, China. SC was immediately placed in paper bags, sealed, and kept in a cooler with ice to maintain freshness. Fresh SCs were cut into 1 cm^3^ pieces and placed in a 50 °C thermostatic air blowing drying oven (GZX-GF101-3-BS-II) (Shanghai, China) for 1 d and then weighed. The weight of dried SC was 56.73 g per 100 g. The dried SC was crushed and sifted with a 0.425 mm mesh. Then, 100 g of dried SC powder was weighed using an electronic balance (BSA224S OLABO) (Beijing, China), soaked in 10 times (v/w) pure water for 1 h, boiled for 1.5 h, and filtered. Eight times (v/w) of purified water Total RNA extraction and RNA-seq library construction was added to the filtrate, boiled for another 1.5 h, and filtered. The two filtrates were merged and evaporated using rotary evaporation (CHRIST RVC 2-25 CD plus) (Shanghai, China) under a vacuum at 60 °C. After further freeze-drying, the volume was kept constant at 5.3 g/ml and stored at 4 °C until further analysis (Yuan et al., 2019).

**Liquid Chromatograph Mass Spectrometer (LC-MS) analysis of SC**

Prior to LC-MS analysis, the solution filtered through 0.22 m membrane filter.

The LC system (Agilent 1290-6460 series) was composed of a binary pump, an autosampler and a column oven. The chromatographic separation was performed at 25 °C on a Zorbax Eclipse Plus C18 column (Agilent, 2.1×50 mm, 2.2mm, Zorbax) with 0.1% (v/v) formic acid in water (A) and methanol (B) as mobile phases, delivered at 0.3mL/min. The gradient elution program was as follows: 0-10 min, 99% A-1% B to 70% A-30% B; 10-20 min, 55% A-45% B; and 20-30 min, 1% A-99% B.

The analytes were detected using MS/MS with an electrospray ionization (ESI)-interface in negative multiple reaction monitoring (MRM)-mode. Mass transitions for SC (m/z 1000-100) were optimized. Quantification was conducted in MRM ion mode. The parameters of the mass spectrometer were optimized and set as follows: HV capillary at 3.5kV, nebulizer at 40 psi, and drying gas flow rate at 10 L/min at 300 °C.

**Total RNA extraction**

We selected three samples from the LQH group with significant hepatic inflammatory changes and no other problems, and three samples from the NC group with clear hepatocytes for transcriptome sequencing. Total RNA was extracted from the livers of plateau zokor rats using TRIzol reagent (Invitrogen Carlsbad, CA, USA) according to the manufacturer’s protocol. RNA integrity was assessed using the RNA Nano 6000 Assay Kit of the Bioanalyzer 2100 system (Agilent Technologies, CA, USA). Sequencing was performed at Beijing Novo Co et al., Ltd. (Beijing, China) (Yang et al., 2015).

**Library preparation for transcriptome sequencing**

Briefly, mRNA was purified from total RNA using poly-T oligo-attached magnetic beads. Fragmentation, cDNA, and library preparation synthesis were then performed (Yang et al., 2015).

**Clustering and sequencing**

Clustering of index-coded samples according to the manufacturer’s instructions (Yang et al., 2015).

**Sequence alignment and functional analysis**

Raw reads in the FASTQ format were processed and cleaned (clean reads) using Trinity (version 2.4.0). The RNA-seq reads in the FASTQ file were mapped to the Upper Galilee mountains blind mole rat (*Nannospalax galili*) reference genome (BioProjects: PRJNA254049). After the raw read data were cleaned, Trinity was used for transcript assembly, Corset (Nadia M Davidson Alicia Oshlack 2014) was used to aggregate redundant transcripts, and the assembled transcripts were evaluated using tblastn, augustus, and hmmer software. Gene function was annotated using the Nr, Nt, Pfam, KOG/COG, Swiss-Prot, KEGG, and GO databases (Kanehisa et al., 2019; Kanehisa et al., 2023). Differential expression analysis was performed using the DESeq2 R package (version 1.20.0) (Yang et al., 2015). We used the native version of GSEA analysis tool http://www.broadinstitute.org/gsea/index.jsp. GSEA was performed using GO, KEGG datasets, respectively. AS is an important mechanism for regulating gene expression and protein changes. AS events were analyzed using rMATS (version 4.1.0) software. PPI analysis of differentially expressed genes was based on the STRING database, which is known and predicts protein-protein interactions.

Xie, J. X.; Lin, G. H.; Liu, C. X.; Yang, C. H.; Deng, X. G.; Cui, X. F.; Li, B.; Zhang, T. Z.; Su, J. P. Diet selection in overwinter caches of plateau zokor (*Eospalax baileyi*). *Acta Theriol.* **2014**, *59*, 337-345.

Yang, M.; Zhu, L. P.; Pan, C.; Xu, L. M.; Liu, Y. L.; Ke, W. D.; Yang, P. F. Transcriptomic analysis of the regulation of rhizome formation in temperate and tropical lotus (*Nelumbo nucifera*). *Sci. Rep*. **2015**, *17*, 13059.

Kanehisa, M. Toward understanding the origin and evolution of cellular organisms. *Protein. Sci*. **2019**, *28*, 1947-1951.

Kanehisa, M.; Furumichi, M.; Sato, Y.; Kawashima, M.; Ishiguro-Watanabe, M. KEGG for taxonomy-based analysis of pathways and genomes. *Nucleic. Acids. Res*. **2023**, *51*, D587-D592.
